# PAIN COMPETENCY EVALUATION IN DEMENTIA (PACED) SCALE: DEVELOPMENT AND EVALUATION OF CONTENT VALIDITY

**DOI:** 10.1093/geroni/igae098.2360

**Published:** 2024-12-31

**Authors:** Yo-Jen Liao, Ying-Ling Jao, Marie Boltz, Nai-Ching Chi, Diane Berish, Terrence Murphy

**Affiliations:** The Pennsylvania State University, University Park, Pennsylvania, United States; Pennsylvania State University, University Park, Pennsylvania, United States; The Pennsylvania State University, University Park, Pennsylvania, United States; University of Iowa, Iowa City, Iowa, United States; The Pennsylvania State University, University Park, Pennsylvania, United States; The Pennsylvania State University, University Park, Pennsylvania, United States

## Abstract

Pain management remains under-addressed among persons with dementia; understanding nurses’ pain management competency in this particular population is a first step. However, limited tools objectively evaluate nurses’ pain management competency among people with dementia. This study aimed to develop Pain Competency Evaluation in Dementia (PACED), an observational scale to evaluate nurses’ pain management competency in those with dementia. Reviews of pain management literature, guidelines, and protocols were conducted and consisted of five sections: history taking, pain assessment, physical and functional assessment, pain treatment, and documentation. Each item is scored on a 0-3 scale (0=not performed, 1=inaccurate, 2=partially accurate, or 3=accurate). This scale was distributed to seven researchers and clinicians with expertise in pain and/or dementia to evaluate its content validity using a Delphi survey approach. Each expert rated each item on a 1-4 scale (1= unclear/irrelevant to 4=very clear/relevant). The index of content validity (I-CVI) was calculated as the proportion of items with ratings of 3 and 4, with higher scores indicating better clarity/relevancy. A relevance I-CVI>0.79 and a clarity I-CVI≥0.83 are considered relevant and clear, respectively. Results showed that 24/24 items are relevant (mean I-CVI=0.97 [SD=0.06]) and 9/24 items are clear (mean I-CVI=0.73 [SD=0.12]). Experts suggested creating a monitoring tool/checklist for nurses, combining similar items, and adding more details for certain items. Further revisions are needed for the 15 unclear items across 5 sections. Psychometric evaluations are essential to validate the PACED scale. Ultimately, the validated PACED scale will help improve pain management in persons with dementia.

